# Report of SARS-CoV-2 B1.1.7 Lineage in Morocco

**DOI:** 10.1128/MRA.00240-21

**Published:** 2021-04-22

**Authors:** Mouna Ouadghiri, Tarik Aanniz, Abdelomunim Essabbar, Myriam Seffar, Hakima Kabbaj, Ghizlane El Amin, Amal Zouaki, Saaïd Amzazi, Lahcen Belyamani, Azeddine Ibrahimi

**Affiliations:** a Medical Biotechnology Laboratory (MedBiotech), Bioinova Research Center, Rabat Medical and Pharmacy School, Mohammed V University, Rabat, Morocco; b Laboratoire Central de Virologie, Centre Hospitalo-Universitaire Ibn Sina, Hôpital des Spécialités, Rabat, Morocco; c Laboratory of Human Pathologies Biology, Faculty of Sciences, Mohammed V University, Rabat, Morocco; d Emergency Department, Military Hospital Mohammed V, Rabat Medical and Pharmacy School, Mohammed V University, Rabat, Morocco; Queens College CUNY

## Abstract

Here, we report the near-complete genome sequence and the genetic variations of a clinical sample of severe acute respiratory syndrome coronavirus 2 (SARS-CoV-2) harboring the N501Y mutation assigned to the B.1.1.7 lineage. The sample was collected from a nasopharyngeal swab of a female patient from Temara, Morocco, and the sequencing was done using Ion S5 technology.

## Abstract (precis)

Here, we report the near-complete genome sequence and the genetic variations of a clinical sample of SARS-CoV-2 harboring the N501Y mutation assigned to the B.1.1.7 lineage. The sample was collected from a nasopharyngeal swab of a female patient from Temara, Morocco, and the sequencing was done using Ion S5 technology.

## ANNOUNCEMENT

A new *Betacoronavirus* strain of the *Coronaviridae* family named severe acute respiratory syndrome coronavirus 2 (SARS-CoV-2) is the etiological agent of coronavirus disease 19 (COVID-19) (1–3). The identification of new mutations may contribute to characterizing the virus, mapping its spread, and better understanding its biological and clinical features (4, 5). In this report, near-whole-genome sequencing (WGS) of SARS-CoV-2 was carried out using Ion S5 sequencing technology to detect new variants (6).

The sampling was carried out on 8 January 2021. RNA was extracted from a nasopharyngeal swab sample of a 48-year-old female from Temara, Morocco, at the Central Laboratory of Virology, Hospital of Specialties of Rabat, using a Maxwell RSC blood DNA extraction kit (Promega, USA). The patient was identified as positive for COVID-19 by reverse transcriptase quantitative PCR using a SARS-CoV-2 kit (MAScIR, Morocco) and exhibited cycle threshold (*C_T_*) values of 19 for both S and RdRp genes. The cDNA was prepared using a SuperScript VILO cDNA synthesis kit (Invitrogen, Thermo Fisher Scientific, USA). A total of 15 ml of cDNA was used to prepare a SARS-CoV-2 library by using an Ion AmpliSeq kit for Chef DL8 (Thermo Fisher Scientific, USA). The library was adjusted to 30 pM and loaded onto the Ion Chef instrument (Thermo Fisher Scientific, USA) for emulsion PCR, enrichment, and loading onto the Ion S5 530 chip. WGS was performed using the Ion AmpliSeq SARS-CoV-2 research panel designed by Thermo Fisher Scientific for complete viral genome sequencing according to instructions for use on an Ion Gene Studio S5 Prime series system.

Raw data were analyzed using Torrent Suite software v 5.12.0. The NGS QC Toolkit v 2.3.3 was used to remove low-quality and short reads. Variant Caller v 5.10.1.19 was used to detect variants compared to the reference genome (Wuhan-Hu-1, GenBank accession number MN908947.3), while the consensus sequence was generated using IRMAreport v 1.3.0.2. The annotation was carried out using COVID19AnnotateSnpEff v 1.3.0.2, a plugin specifically developed for SARS-CoV-2. Default parameters were used for all software (7).

Our analysis allowed us to obtain a near-complete SARS-CoV-2 genome of 29,805 bp length with an average read length of 206 bp and an overall DNA G+C content of 37.98%. From 879,763 reads, 862,414 reads were correctly mapped, covering 97.56% of the total genome with a mean depth of 5,726×.

The genetic variant process revealed a total of 34 variations, including 15 in open reading frame 1ab (ORF1ab; 7 synonymous variants, 6 missense variants, 1 conservative in-frame deletion, and 1 upstream gene variant), 9 in spike genes (7 missense variants and 2 disruptive in-frame deletions), 3 in ORF8 (2 missense variants and 1 stop gained), and 7 in the N gene (6 missense variants and 1 synonymous variant). The spike gene carries the mutation known as N501Y (Asn501Tyr; c.1501A>T). This mutation cooccurs with several mutations, including missense mutations (A570D, P681H, T716I, S982A, and D1118H), as well as disruptive in-frame deletions (H69-V70 and Y145) (8, 9). The genomic features of the sequenced sample are summarized in Table 1. The phylogenetic analysis using Phylogenetic Assignment of Named Global Outbreak (PANGO) lineages (10) revealed that the strain belongs to the B1.1.7 lineage.

**TABLE 1:** Types and effects of identified gene variations compared to the reference strain, Wuhan-Hu-1 (GenBank accession number MN908947.3)

Gene	Nucleotide position	Nucleotide change	Residue change	Effect
ORF1ab	241	c.–25C>T	No change assigned	Upstream gene variant
	913	c.648C>T	p.Ser216Ser	Synonymous variant
	3037	c.2772C>T	p.Phe924Phe	Synonymous variant
	3267	c.3002C>T	p.Thr1001Ile	Missense variant
	5388	c.5123C>A	p.Ala1708Asp	Missense variant
	5986	c.5721C>T	p.Phe1907Phe	Synonymous variant
	6954	c.6689T>C	p.Ile2230Thr	Missense variant
	10277	c.10012C>T	p.Leu3338Phe	Missense variant
	11287	c.11023_11031delTCTGGTTTT	p.Ser3675_Phe3677del	Conservative in-frame deletion
	14408	c.14144C>T	p.Pro4715Leu	Missense variant
	14676	c.14412C>T	p.Pro4804Pro	Synonymous variant
	14925	c.14661C>T	p.Val4887Val	Synonymous variant
	15279	c.15015C>T	p.His5005His	Synonymous variant
	16176	c.15912T>C	p.Thr5304Thr	Synonymous variant
	17615	c.17351A>G	p.Lys5784Arg	Missense variant
S	21764	c.204_209delACATGT	p.His69_Val70del	Disruptive in-frame deletion
	21990	c.432_434delTTA	p.Tyr145del	Disruptive in-frame deletion
	23063	c.1501A>T	p.Asn501Tyr	Missense variant
	23271	c.1709C>A	p.Ala570Asp	Missense variant
	23403	c.1841A>G	p.Asp614Gly	Missense variant
	23604	c.2042C>A	p.Pro681His	Missense variant
	23709	c.2147C>T	p.Thr716Ile	Missense variant
	24506	c.2944T>G	p.Ser982Ala	Missense variant
	24914	c.3352G>C	p.Asp1118His	Missense variant
ORF8	27972	c.79C>T	p.Gln27^*a*^	Stop gained
	28048	c.155G>T	p.Arg52Ile	Missense variant
	28111	c.218A>G	p.Tyr73Cys	Missense variant
N	28280	c.7G>C	p.Asp3His	Missense variant
	28281	c.8A>T	p.Asp3Val	Missense variant
	28282	c.9T>A	p.Asp3Glu	Missense variant
	28881	c.608G>A	p.Arg203Lys	Missense variant
	28882	c.609G>A	p.Arg203Arg	Synonymous variant
	28883	c.610G>C	p.Gly204Arg	Missense variant
	28977	c.704C>T	p.Ser235Phe	Missense variant

a A stop codon.

### Data availability.

This sequence was deposited in the GenBank and GISAID databases under the accession numbers MW803167 and EPI_ISL_1137621, respectively. The raw reads were deposited in the NCBI Sequence Read Archive (SRA) under the accession number SRR13811335.
